# Clinical Characteristics, Medication Use, and Short-Term Outcomes in Patients With Rheumatoid Arthritis: A Prospective Observational Study at a Tertiary Care Centre in Central India

**DOI:** 10.7759/cureus.84785

**Published:** 2025-05-25

**Authors:** Keshao B Nagpure, Asit Kumar Parida, Sunita Kumbhalkar, Amol Dube, Ishan Verma

**Affiliations:** 1 General Medicine, All India Institute of Medical Sciences, Nagpur, Nagpur, IND; 2 General Medicine, LN Medical College and Research Center, Bhopal, IND

**Keywords:** clinical profile, comorbidities, disease activity score (das-28), disease-modifying antirheumatic drugs (dmards), extraarticular manifestation, methotrexate, rheumatoid arthritis, short term outcomes, treatment patterns

## Abstract

Background

Rheumatoid arthritis (RA) is a chronic inflammatory arthritis associated with articular and extraarticular manifestations. Clinical profile, treatment patterns, and treatment outcome of RA patients vary based on geographic location, both globally and among certain ethnic groups within a country.

Objectives

We aim in this study to describe the demographic and clinical characteristics, treatment patterns, and short-term treatment outcomes by DAS-28 CRP score (disease activity score) in patients with RA in Central India.

Methods

This prospective observational study was conducted from September 2023 to September 2024 at a tertiary care hospital in central India. A total of 121 RA patients aged over 18 years, diagnosed as per the 2010 American College of Rheumatology/European League Against Rheumatism (ACR/EULAR) criteria or already receiving treatment for RA, were enrolled. Data on demographics, clinical profiles, drug usage patterns, comorbidities, and treatment outcomes were collected using a structured case record form over a follow-up period of three months.

Results

Out of 121 total participants, 101 (83.5%) were females. The female-to-male ratio was 5:1. The participants' mean age was 46.99±12.84 years. Of the 121 patients, 75 (61.9%) were previously diagnosed with RA, with 32 (42.7%) of them having disease duration between 1 and 5 years (mean duration: 4.1 years). Common symptoms included arthralgia (118 [97.5%]), early morning stiffness (102 [84.3%]), and weight loss (45 [37.2%]). Polyarticular involvement was seen in 105 (86.8%) of patients, most commonly affecting the hand joints. Boutonniere and Swan neck deformities were present in 18 (14.9%) and 16 (13.2%) of cases, respectively. Anemia (65 [53.7%]) was the most frequent extra-articular manifestation. Common comorbidities included obesity in 38 (31.4%), hypertension in 27 (22.3%), and ILD/COPD in 18 (14.9%) of the patients. Methotrexate and folic acid were each used in 103 (85.1%) patients, with hydroxychloroquine in 86 (71.1%); while 75 (61.9%) received steroids.

Conclusions

RA is predominant among the middle-aged population and females. Arthralgia and early morning stiffness in hand joints were the most common articular symptoms, while anemia was the most common extraarticular feature. Obesity followed by hypertension was the most common comorbidity reported in our study. Dual DMARDs (disease-modifying antirheumatic drugs) therapy, particularly the methotrexate-hydroxychloroquine combination, was most preferred. Treatment with DMARDs led to a significant reduction in disease activity over three months. These findings highlight RA characteristics and treatment patterns in Central India.

## Introduction

Rheumatoid arthritis (RA) is the most common form of chronic inflammatory arthritis characterized by symmetric, inflammatory erosive polyarthritis predominantly involving peripheral joints like small joints of hands and feet. RA affects ~0.5-1% of the adult population worldwide. RA occurs more commonly in women than in men, with a ratio of 2-3:1 [1]. In India, the prevalence of RA is estimated to be about 0.75% [2]. RA presents with a wide variety of clinical presentations involving both articular and extraarticular manifestations. Persistently active RA often results in articular cartilage and bone destruction, which may lead to disability and deformity of joints with diminished quality of life among affected individuals. Extra-articular manifestations may develop during the clinical course of RA.

The 2010 American College of Rheumatology and the European League Against Rheumatism (ACR/EULAR) classification criteria were developed to facilitate early diagnosis and initiation of disease-modifying antirheumatic drugs (DMARDs) in patients at risk of developing persistent or erosive disease [3]. Diagnosis of RA is mainly clinical (signs and symptoms of chronic inflammatory arthritis) supported by acute phase reactants (erythrocyte sedimentation rate [ESR] and C-reactive protein [CRP]) and serology (RA factor and anti-citrullinated peptide antibodies [ACPA]). Prognostic factors, including positive autoantibodies, early bone erosions, extra-articular involvement, and coexisting comorbidities, guide treatment decisions and predict disease severity. Modern treatment strategies, including the treat-to-target (T2T) approach, emphasize early, aggressive therapy aimed at achieving remission or low disease activity. Tools such as the Disease Activity Score-28 with CRP (DAS28-CRP), Clinical Disease Activity Index (CDAI), and Simplified Disease Activity Index (SDAI) are commonly used in clinical practice to monitor treatment response and adjust therapy accordingly [4].

RA exhibits geographic and ethnic variability in clinical profile, treatment patterns, and outcomes [5,6]. Despite advances in understanding its pathogenesis and treatment, a significant lack remains in the knowledge of its demographic patterns, clinical characteristics, pattern of drug use, and real-world outcomes in the setting of Indian populations. Therefore, this prospective observational study aimed to describe the demographic characteristics, clinical characteristics, pattern of medication use, and short-term treatment outcomes by DAS-28 CRP score (Disease Activity Score) in patients with RA attending a tertiary care center in central India.

## Materials and methods

Study design and duration

A prospective observational study was conducted from September 2023 to September 2024 (12 months).

Study setting

Patient recruitment was done at the General Medicine OPD (Outpatient Department) and Rheumatology OPD & General Medicine wards of All India Institute of Medical Sciences, Nagpur, Maharashtra (Central India).

Study participants

All confirmed cases of rheumatoid arthritis (RA) according to 2010 ACR/EULAR criteria for RA diagnosis or previously diagnosed cases of rheumatoid arthritis taking treatment and age more than 18 years. Patients with a flare of RA were also included in the study. Patients with undifferentiated arthritis, overlap syndrome, and coexisting connective tissue disease like systemic lupus erythematosus were excluded from the study. The research protocol was approved by the Institute Research Cell, followed by the Institute Ethics Committee (IEC). All enrolled participants were adequately informed about the study, and written informed consent was obtained prior to their inclusion.

Data collection

Data was collected in case record form (CRF), which was completed by patients and physicians, capturing demographic profile, clinical profile, laboratory profile, pattern of drug use, comorbidities & outcome at each month up to three months.

Outcomes and measures

The demographic profile collected included age, gender, residence, education, marital status, religion, occupation, socio-economic status, and body mass index (BMI). Residences are grouped into rural & urban. Education was categorized into seven categories: illiterate, primary school, middle school, high school, intermediate/diploma, graduate, and postgraduate. The patients' religions were categorized into Hindu, Muslim, Christian, Sikh, Buddhist, and others. Marital status was categorized as unmarried, married, separated, and widow/widower. Occupations of the patients were divided among professionals, skilled labor, general labor, housewives, farmers, police/military, business, clerks, disability, and any other specific occupation. The Kuppuswamy Socioeconomic Status (SES) scale was used to classify patients into five socioeconomic classes: upper, upper-middle, lower-middle, upper-lower, and lower [7]. The weight and height of each patient were noted and body mass index (BMI) was calculated in (kg/m²). Obesity was defined as per the revised Indian consensus statement for the diagnosis of obesity for the Indian population. Normal BMI: 18-22.9 kg/m², overweight:23-24.9 kg/m², obesity: ≥25 kg/m², underweight: ≤23 kg/m2 [8]. Disease duration (years/months) was noted at the time of presentation, and patients were classified into newly diagnosed & previously diagnosed patients.

Detailed clinical history was noted regarding the onset, duration, and progress of the articular and extraarticular symptoms. Detailed musculoskeletal and systemic examinations were done and noted in the CRF. All recent and old investigations were noted: complete blood count, ESR, CRP, RA factor, and ACPA. ESR was calculated on ‘Westergren’s Pipette,’ and 15 mm/hr was taken as the cutoff as per the normal laboratory range. CRP was calculated by ‘dry chemistry,’ and 5 mg/L was set as the cutoff as per laboratory reference. RF was measured by the ‘latex agglutination test,’ and 14 IU/ml was taken as the cutoff. ACPA was measured by ‘chemiluminescence assay,’ and 17 U/ml was set as the cutoff as per laboratory reference.

Drug pattern was assessed by the type of drugs used, like non-steroidal anti-inflammatory drugs (NSAIDs), DMARDs, steroids, and biologicals, and their doses, duration, and side effects. Disease activity was assessed by DAS28-CRP score in every patient by assessing tender joint count, swollen joint count, a visual analog scale (VAS) of the patient's general health (VAS-GH scored 0-100), and CRP level (milligram/liter) by using the software. The level of disease activity can be interpreted as remission (DAS28 < 2.6), low (2.6 ≤ DAS28 < 3.2), moderate (3.2 ≤ DAS28 ≤ 5.1), or high (DAS28 >5.1) [9]. Patients were followed every month for up to three months, and the disease activity score (DAS 28 score) was assessed at each visit. The outcome of treatment was assessed by disease activity with the DAS-28 CRP score at each visit.

Statistical analysis

Data collected from the case record form was updated in the master chart and inserted into the Excel sheet (Redmond, USA). Statistical analysis by STATA software (version 17). Continuous variables, such as age and duration of the disease, were expressed as mean ± standard deviation (S.D.) and/or median (IQR). Categorical variables were expressed as percentages and absolute frequencies. To assess the association between categorical variables and groups, we used the chi-square test or Fisher’s exact test. For comparing a quantitative variable between groups, we used the t-test if the data is normally distributed, and variance assumptions are met or the Wilcoxon Rank-Sum test if these assumptions are violated. p<0.05 was considered statistically significant for all the analyses.

## Results

A total of 121 patients with rheumatoid arthritis (RA) were enrolled in the study and followed monthly for up to three months; at the end of one month, six patients were lost to follow-up. A total of 101 (83.5%) patients were females and 20 (16.5%) patients were males. The male-to-female ratio was 1:5. The participants' mean age was 46.99 (SD ±12.84; 95% CI: 44.7-49.28). The mean age of male participants was 49.90 years (SD ±18.78; 95% CI: 41.67-58.13), while the mean age of female participants was 46.42 years (SD ±11.35; 95% CI: 44.20-48.64) (Table 1).

**Table 1:** Age distribution of patients with rheumatoid arthritis

Variables	Mean Age ± SD (Years)
Males (n=20)	49.90±18.78
Females (n=101)	46.42±11.35
Total Patients (n=121)	46.99±12.84

The most common age group was 50-59 years (32.23%), followed by 40-49 years (27.27%). Half of the patients were from rural areas (50.4%), and the rest of them were from urban areas (49.6%). The majority of patients (86.0%) included Hindus; Muslims and Buddhists were equal in proportion, i.e., 5.8%. Most of the patients (84.3%) were married. Most of the patients were educated up to the high school level (28.1%), followed by primary school education (20.7%), middle school (14%), graduate school (13.2%), and intermediate/diploma (10.7%). A total of 11 (9.1%) patients were illiterate, and only 4.1% were postgraduates. Most of the patients were housewives (55.4%), followed by farmers (14.9%). As per the Modified Kuppuswamy scale, the majority of the patients, 44 (36.4%), were from the lower middle class, and 43 (35.5%) were from the upper lower class. We classified our patients as per the revised consensus statement for the diagnosis of obesity in the Indian population [8]. Among a total of 121 patients, 38 (31.40%) were obese, 20 (16.53%) were overweight, 12 (9.92%) were underweight, and the majority of patients, i.e., 51 (42.15%), had a normal BMI. A total of 10 (8.26%) patients had a smoking habit, 22 (18.18%) patients used tobacco, and 9 (7.44%) patients had a history of alcohol consumption. A total of 16 (13.22%) patients had their first-degree relatives with RA, and the rest 105 (86.78%) patients had no family history of RA. A total of 61.99% of patients were previously diagnosed with RA, and the rest 38.01% were newly diagnosed in our tertiary care institute. Of the 121 RA patients studied, most had a disease duration of less than one year, including all 46 newly diagnosed cases. Additionally, 32 patients (26.44%) had a duration of 1-5 years, 28 (23.14%) had 5-10 years, and 10 (8.26%) had more than 10 years. The mean disease duration was 4.1 years (Table 2).

**Table 2:** Baseline sociodemographic characteristics of the rheumatoid arthritis patients

Sociodemographic Characteristics	Frequency (n=121)	Percentage (%)
Age group(years)		
18-29	12	9.92
30-39	21	17.36
40-49	33	27.27
50-59	39	32.23
60-69	11	9.09
70-79	3	2.48
> 80	2	1.65
Gender		
Male	20	16.53
female	101	83.46
Residence		
Rural	61	50.4
Urban	60	49.6
Religion		
Hindu	104	86
Muslim	7	5.8
Buddhist	7	5.8
Sikh	2	1.7
Christian	1	0.8
Marital status		
Married	102	84.3
Unmarried	14	11.6
Widow/widower	4	3.3
Separated	1	0.8
Education		
Postgraduate/honours	5	4.1
Graduate	16	13.2
Intermediate/Diploma	13	10.7
High School	34	28.1
Middle School	17	14
Primary School	25	20.7
Illiterate	11	9.1
Occupation		
Professionals	12	9.9
Business	2	1.7
Skilled Labor	6	5
General Labor	10	8.3
Farmer	18	14.9
Housewife	67	55.4
Unemployed	6	5
Socioeconomical status (Class)		
Lower	9	7.4
Upper lower	43	35.5
Lower middle	44	36.4
Upper middle	24	19.8
Upper	1	0.8
Body mass index (Kg/m^2^)		
Underweight (<18)	12	9.92
Normal (18-22.9)	51	42.15
Overweight (23-24.9)	20	16.53
Obese (>25)	38	31.40
History of Addiction		
Smoking	10	8.26
Smokeless Tobacco(chewing)	22	18.18
Alcohol	9	7.44
Family history (First degree relatives with RA)		
yes	16	13.22
No	105	86.78
Diagnosis status		
Newly diagnosed	46	38.01
Previously Diagnosed	75	61.99
Disease Duration		
0-1 year	51	42.15
1-5 years	32	26.44
5-10 years	28	23.15
>10 years	10	8.26

In this study, the most prevalent clinical feature was arthralgia, observed in 97.5% of RA patients, followed by early morning stiffness in 84.3%. Arthritis was noted in 80% of the patients. Other clinical features included weight loss in 37.2%, deformities in 28.92%, fatigue in 28.09%, and fever in 10.74% of the patients. A total of 86.77% of patients had polyarticular RA, 12.40% had oligoarticular RA, and only 0.8% had a monoarticular pattern. The most common joint involved was the wrist joint in 88.4%, followed by the small joints of the hands (proximal interphalangeal joint/metacarpophalangeal joint) in 83.5%. Either elbow or shoulder or both joint involvement was seen in 74.4% of patients; 67.8% of the patients had either knee or hip involvement, 35.5% of patients had ankle involvement, and 22.5% of patients had metatarsophalangeal joint involvement. Cervical and temporomandibular joint involvement was seen in the least number of patients, i.e., 9.2%. Among 121 patients, boutonniere deformity was found in 14.9% of patients, followed by swan neck deformity in 13.2%, 11.6% had Z deformity, 6.6% had subluxation of the metacarpal or metatarsal heads, 5.8% had ulnar deviation, and 3.3% had hallux valgus (Table 3).

**Table 3:** Clinical characteristics of the rheumatoid arthritis patients PIP: Proximal interphalangeal joint, MCP: Metacarpophalangeal joint, MTP: Metatarsophalangeal joint, TMJ: Temporomandibular joint, ESR: Erythrocyte sedimentation rat, CRPP: C-reactive protein

Clinical characteristics	Frequency (n=121)	Percentage (%)
Common clinical feature		
Fever	13	10.74
Fatigue	34	28.09
Weight loss	45	37.2
Arthralgia	118	97.52
Arthritis	97	80.16
Early morning stiffness	102	84.3
Deformities	35	28.92
Pattern of joint involvement		
Monoarticular RA (1 joint)	1	0.8
Oligoarticular RA (2-4 joints)	15	12.40
Polyarticular RA (> 5 Joints)	105	86.77
Types of Joint involvement		
Wrist	107	88.4
PIP/MCP	101	83.5
Elbow/Shoulder	90	74.4
Ankle/Subtalar joint	43	35.5
Knee/Hip	82	67.8
MTP/PIP	27	22.5
Cervical/TMJ	11	9.2
Types of Deformities		
Swan Neck	16	13.2
Boutonniere Deformity	18	14.9
Z Deformity	14	11.6
Ulnar Deviation	7	5.8
Eversion of subtalar joint	1	0.8
Hallux Valgus	4	3.3
Subluxation of Metatarsophalangeal /Metacarpophalangeal Heads	8	6.6
Extra-articular Manifestations	92	76
Anemia	65	53.72
Alopecia	22	18.18
Musculoskeletal	20	16.53
Neuropathy	19	15.70
Thrombocytosis	19	15.70
Interstitial Lung Disease	15	12.4
Dermatological/Purpura	13	10.74
Rheumatoid Nodules	12	9.92
Scleritis/Episcleritis	12	9.92
Serositis	9	7.44
Cardiac Manifestation	9	7.44
Secondary Sjogren syndrome	6	4.96
Raynaud’s phenomenon	4	3.31
Vasculitis	4	3.31
Inflammatory Markers		
Raised ESR	110	90.1
Raised CRP	101	83.47

Extra-articular manifestations were observed in 76% of RA patients, with anemia being the most common, present in 53.72%. Alopecia was seen in 18.18% of patients, while 15.70% had thrombocytosis. Musculoskeletal manifestations-including osteoporosis, osteoarthritis, and myalgia-were reported in 15.70%, and an equal proportion (15.70%) had neuropathy (mixed sensory-motor neuropathy, pure sensory neuropathy, carpal tunnel syndrome, or mononeuritis multiplex). Interstitial lung disease was found in 12.40%, dermatological involvement such as purpura or pyoderma gangrenosum in 10.74%, scleritis or episcleritis in 9.92%, and rheumatoid nodules in 9.92% of patients. Serositis and cardiac manifestations (cardiomyopathy, heart failure, aortic root abnormalities, and arrhythmia) were each observed in 7.44% of patients. Obesity was the most commonly observed (31.4%) comorbidity, followed by hypertension (22.31%) and interstitial lung disease (14.87%). Diabetes mellitus and cardiovascular diseases were observed in 11.57% of patients. Depression and hypothyroidism were present in 10.74% each. Stroke was reported in 4.13%, tuberculosis in 7.44%, malignancy in 2.48%, chronic liver disease in 6.61%, and chronic renal failure in 5% of patients (Table 4).

**Table 4:** Comorbidities in rheumatoid arthritis patients

Comorbidities	Frequency (%) (n =121)
Obesity	38 (31.40%)
Hypertension	27 (22.31%)
Diabetes mellitus	14 (11.57%)
Cardiovascular Diseases	14 (11.57%)
Chronic Obstructive Pulmonary Disorder (COPD)	18 (14.87%)
Hypo/Hyperthyroidism	13 (10.74%)
Depression	13 (10.83%)
Tuberculosis	9 (7.44%)
Chronic Liver Disease	8 (6.61%)
Chronic Renal Failure	6 (5%)
Stroke	5 (4.13%)
Malignancy	3 (2.48%)

**Figure 1:**
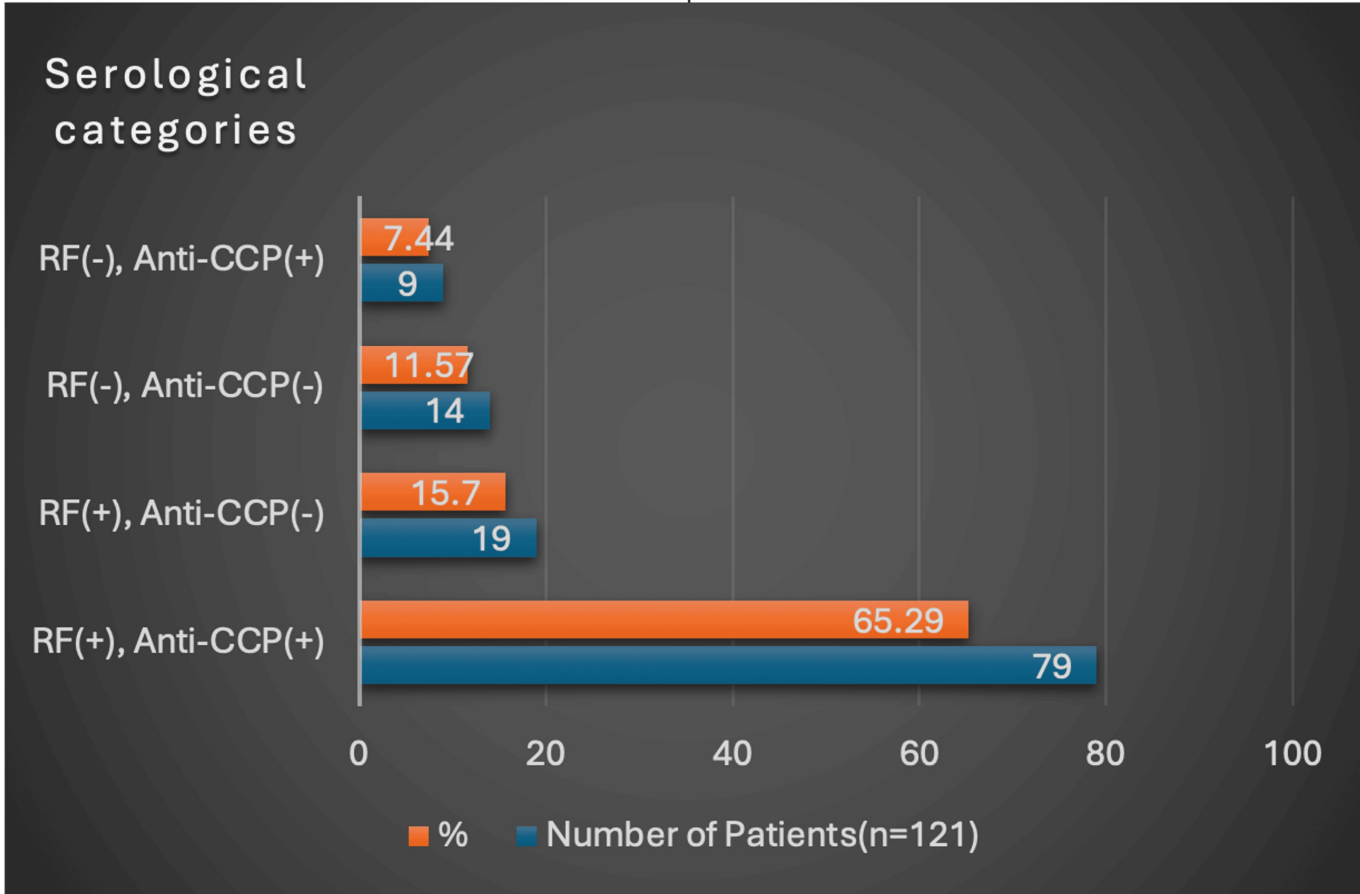
Serological categories of rheumatoid arthritis patients RF: RA factor, Anti-CCP: Anti-cyclic citrullinated peptide (antibodies). The image is created by the author.

Elevated ESR was observed in 90.91% of patients, while 83.47% had raised CRP levels, indicating a high degree of inflammatory activity. The median ESR was 38 mm in the first hour (interquartile range [IQR]: 25-50), and the median CRP level was 25 mg/L (IQR: 7.20-61.50). In this study, 81% of patients were rheumatoid factor (RF) positive, while 72.73% were anti-CCP positive. The median RA factor was 65 IU/mL with an interquartile range (IQR) of 15-208 IU/mL. The median anti-CCP antibody level was 98 U/mL with an interquartile range (IQR) of 10-323 U/mL. Patients were categorized based on RF and anti-CCP status. Overall, 88.43% were positive for either RF or anti-CCP, while 11.57% were negative for both, representing seronegative rheumatoid arthritis (Figure 1).

At the time of presentation, most rheumatoid arthritis patients had moderate disease activity (62.67%), with a slightly higher proportion among newly diagnosed patients (73.91%) compared to those previously diagnosed (62.67%). High disease activity was observed in 23.14% of patients-21.74% of newly diagnosed and 24.00% of previously diagnosed individuals. Low disease activity was less common, seen in 7.44% of patients overall, including 4.35% of newly diagnosed and 9.33% of previously diagnosed patients. Remission was observed in only 2.48% of all patients and was limited to 4.00% of the previously diagnosed group (Table 5).

**Table 5:** Disease activity (DAS28-CRP) on presentation DAS28-CRP: Disease activity score 28-C-reactive protein

Disease activity on presentation	No. of patients (n=121)	Newly diagnosed patients (n=46)	Previously diagnosed patients (n=75)
Remission (<2.6)	3 (2.48%)	0 (0.00%)	3 (4.00%)
Low (2.6-3.2)	9 (7.44%)	2 (4.35%)	7 (9.33%)
Moderate (3.2-5.1)	81 (66.94%)	34 (73.91%)	47 (62.67%)
High (>5.1)	28 (23.14%)	10 (21.74%)	18 (24.00%)

We analyzed the medication patterns among patients, based on drug use recorded across all follow-up visits. Some previously diagnosed patients were either not on DMARDs at baseline or had discontinued them. Two newly diagnosed patients were initially managed with nonsteroidal anti-inflammatory drugs (NSAIDs) or steroids alone. Over the three-month follow-up period, 20.66% of patients had been using alternative medications, which were discontinued, and conventional DMARDs were initiated.

**Figure 2:**
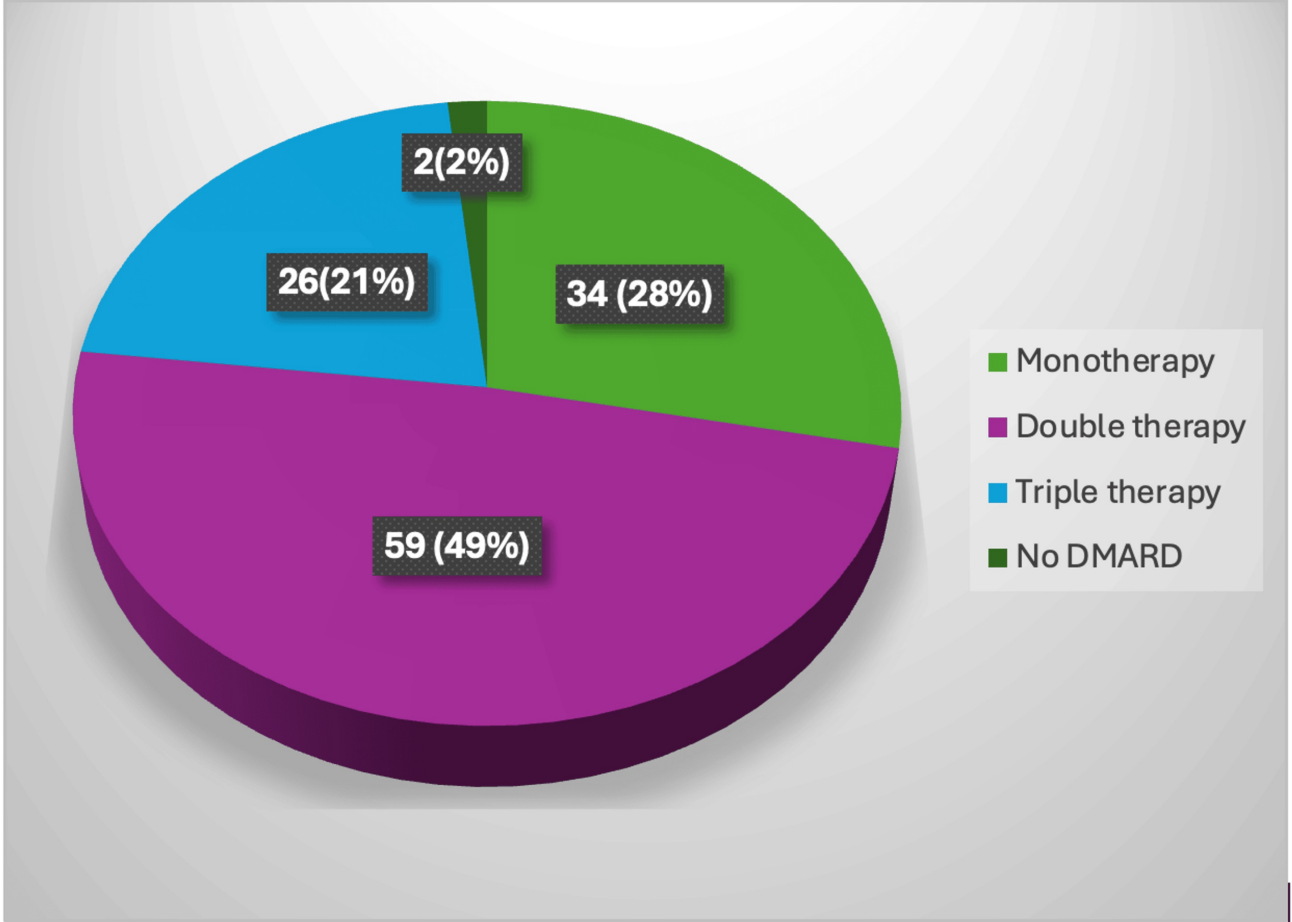
Distribution of conventional synthetic DMARD therapy used by rheumatoid arthritis patients (n=121) DMARDs: Disease-modifying anti rheumatic drugs. The image is created by the author.

Methotrexate was the most commonly prescribed DMARD, used by 85.12% of patients, followed by hydroxychloroquine (HCQ) in 72.72%, sulfasalazine in 29.75%, and leflunomide in 7.44%. Biologic agents such as etanercept, rituximab, or tofacitinib were administered to 8.26% of patients. Steroids were prescribed to 62.80% of the patients, whereas 53.72% received NSAIDs. Additionally, 4.13% of patients were treated with cyclophosphamide. All the patients on methotrexate therapy were on folic acid supplementation. 70.24% of patients were receiving daily calcium plus vitamin D supplementation (Table 6). Among NSAIDs, indomethacin was the most commonly prescribed, followed by naproxen and etoricoxib. Prednisolone is the most commonly used steroid. The average dose of methotrexate was 12.26 mg/week. Among methotrexate users, 35.92% of patients were each on 10 mg/week and 15 mg/week, 18.44% of patients were on 7.5 mg/week, 5.8% were on 20 mg/week, 2% of patients were each on 12.5 mg/week and 25 mg/week. An average dose of hydroxychloroquine, sulfasalazine, and leflunomide was 309.30 mg/day, 1.1 g/day, and 16.25 mg/day, respectively. 98.35% of the patients were receiving conventional synthetic DMARDs (csDMARDs) in various combinations. The most common treatment regimen was double therapy, used by 48.76% of patients, with methotrexate as the primary anchor drug. Monotherapy was prescribed in 28.09% of patients, most commonly with methotrexate. Triple therapy was used in 21.49% of patients, with the combination of methotrexate, hydroxychloroquine, and sulfasalazine being the most frequent (Figure 2).

**Table 6:** Pattern of overall drug use in rheumatoid arthritis patients

Name/Class of Drugs	Number of patients (%) (n=121)
Nonsteroidal-inflammatory drugs (NSAIDs)	58 (47.93%)
Steroids	75 (61.98%)
Methotrexate	103 (85.12%)
Hydroxychloroquine	86 (71.07%)
Sulfasalazine	35 (28.92%)
Leflunomide	8 (6.61%)
Iguratimod	1 (0.82%)
Biological	10 (8.26%)
Alternate medications	25 (20.66%)
Folic acid	103 (85.12%)
Calcium & Vitamin D	85 (70.24%)
Cyclophosphamide	5 (4.13%)

**Figure 3:**
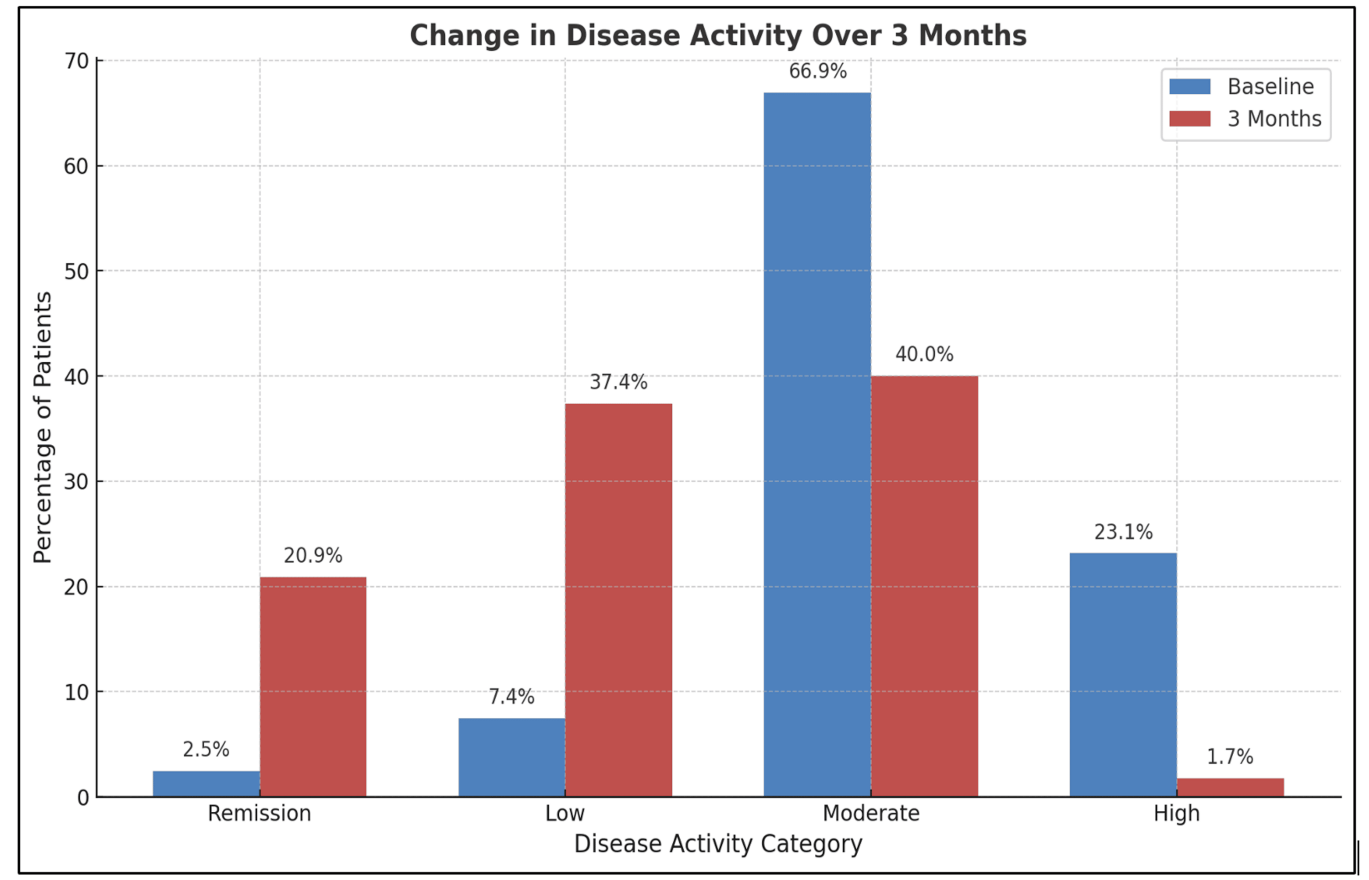
Short-term outcome in patients with rheumatoid arthritis The image is created by the author.

At baseline, 2.48% of patients were in remission, 7.44% had low disease activity, 66.94% had moderate disease activity, and 23.14% had high disease activity. By the end of three months, there was a marked improvement in disease activity. The proportion of patients with high disease activity declined sharply to 1.74%, remission rates increased to 20.87%, and low disease activity rose to 37.39%. Moderate disease activity decreased to 40%. The most notable change was observed in high disease activity, which declined sharply from 23.14% at baseline to 1.74% by the third month (Figure 3).

## Discussion

Rheumatoid arthritis (RA) represents an important public health problem due to its increasing prevalence and associated economic costs. Its importance is underlined by the World Health Organization, which recognizes RA as a disability cause in the world, affecting approximately 1% of the world’s adult population [10]. RA represents a complex interplay of genetic, environmental, and psychosocial factors, leading to significant morbidity and impaired quality of life for affected individuals. The heterogeneity of RA necessitates a nuanced understanding of the demographic characteristics that influence disease manifestation and progression. Notably, factors such as gender, age groups, residential status, marital status, and education levels serve as pivotal components in comprehensively assessing RA patients and tailoring effective management strategies.

The present study aimed to evaluate the clinical profile, treatment patterns, and treatment outcomes of 121 patients diagnosed with rheumatoid arthritis (RA). The majority of patients were female (83.46%), with a female-to-male ratio of 5:1. The overall mean age of participants was 46.99 years. The mean age of female patients was 46.42 years, while that of male patients was 49.90 years. The most frequently affected age group was 50-59 years, followed by 40-49 years. These findings are consistent with existing literature indicating that RA predominantly affects individuals in the middle age range (40 to 60 years). Penserga et al. reported a similar trend in a study involving 266 Filipino patients, with a mean age of 44 years and a marked female predominance [11]. Likewise, Bawazir et al. documented a mean age of 49.7 years, with 85% of patients being female [12]. In an Indian cohort, Tembe et al. also observed a female predominance (88.5%) and typical disease onset between the third and fifth decades of life [13].

Although the exact etiology of rheumatoid arthritis (RA) remains incompletely understood, both genetic and environmental factors are known to contribute to its pathogenesis. A positive family history is an important indicator of genetic susceptibility. In the present study, 13.22% of patients reported having at least one first-degree relative affected by RA, a proportion comparable to that reported by Tembe et al. and Buckman et al. [13,6].

The educational background of the participants was heterogeneous. Specifically, 28.1% had completed high school, 28.0% had pursued some form of higher education, and 44.8% had not attained education beyond middle school; 9.1% had no formal education. The predominance of lower educational attainment in the cohort may have implications for disease outcomes and treatment adherence. Assessing educational status is therefore essential to inform targeted interventions aimed at improving patient education and self-management in rheumatoid arthritis [14]. The socioeconomic status (SES) of the study population was assessed using a composite index incorporating income level, occupation, and education. Among the cohort, 43 % were classified as low SES, 56 % as middle SES, and only 1% of patients were upper SES. Our cohort differs from the cohort studied by Konda et.al where most of the patients (31 %) were from upper SES, 35% of patients were from middle SES, and 34% of patients were from lower SES [15]. This difference can be explained by geographical variations where most of the cohort have lower education, lower income, and were housewives. Use of smokeless tobacco was more common than smoking or alcohol consumption among patients, reflecting regional and gender-related patterns. The low prevalence of smoking in the cohort is likely due to the predominance of female participants, as tobacco smoking is less common among Indian women, whereas smokeless tobacco use is relatively more accepted.

In our cohort, 62% of patients were referred from primary and secondary care with a prior RA diagnosis, while 38% were newly diagnosed at our tertiary center. Early RA (duration <1 year) accounted for 42.15% of cases. The mean disease duration was 4.1 years, lower than Miura et al., who reported 151 months [16]. This disparity likely reflects our inclusion of newly diagnosed patients and earlier referrals in our cohort. Early diagnosis enables timely DMARD initiation, reducing long-term joint damage and disability and emphasizing early rheumatologic referral [17].

The most common clinical feature in our cohort was arthralgia and early morning stiffness, followed by arthritis and constitutional symptoms. The most common joint involved was the wrist joint, followed by either the proximal interphalangeal joint (PIP) or the metacarpophalangeal joint (MCP) or both. The clinical features observed in the present study were comparable to those reported by Penserga et al. [11]. Among all the joint deformities, boutonniere deformity was the most common in our patients, followed by swan neck and Z-deformity. Extra-articular manifestations were observed in 76% of patients, with anemia being the most prevalent. This frequency is higher than that reported in South American populations but comparable to findings from West Asian cohorts [18,19]. The increased prevalence of extra-articular manifestations in the present study may be attributed to the high frequency of anemia, consistent with findings from a previous Indian study [20].

The interaction between rheumatoid arthritis (RA) and comorbidities requires special attention in the management of RA, as comorbid conditions may contribute to a reduced life expectancy in patients with RA. In the present study, comorbidities were identified in 66.9% of patients, representing a substantial proportion of the cohort. Obesity (31.4%) emerged as the most prevalent comorbidity, followed by hypertension, pulmonary diseases (including chronic obstructive pulmonary disease and interstitial lung disease), cardiovascular diseases, diabetes mellitus, hypothyroidism, and depression. These findings are consistent with the Karnataka Rheumatoid Arthritis Comorbidity (KRAC) study conducted by Chandrashekara et al. [21] in South India, which reported hypertension (20.7%), diabetes mellitus (14.4%), and thyroid disorders (18.3%) as the most common comorbid conditions, followed by pulmonary and cardiovascular diseases, as well as psychiatric disorders such as depression. Notably, obesity was not classified as a comorbidity in most previous studies. However, Buckman et al. [6] reported a higher prevalence of obesity (46.4%) in their cohort, with hypertension also featuring prominently among comorbidities. These variations highlight the need for standardized definitions and comprehensive evaluation of comorbidities in patients with rheumatoid arthritis.

In this study, many newly and some previously diagnosed patients were initially using alternative therapies, which were discontinued following counseling on disease chronicity, after which DMARDs were initiated and treatment patterns as well as outcomes were evaluated over a three-month follow-up. Methotrexate (85%) was the most common DMARD used, followed by hydroxychloroquine, sulfasalazine, and leflunomide. Most patients receiving conventional synthetic DMARDs (csDMARDs) were managed with combination therapy, most frequently a regimen comprising methotrexate and hydroxychloroquine. Monotherapy, primarily with methotrexate, was employed less frequently. A subset of patients was treated with triple therapy, with the combination of methotrexate, hydroxychloroquine, and sulfasalazine being the most utilized. The pattern of csDMARD utilization observed in our cohort contrasts with findings from a retrospective study conducted in Colombia, where csDMARDs were underutilized and leflunomide emerged as the most prescribed agent [22]. In contrast, studies from Ghana and central India have identified methotrexate as the preferred csDMARD in routine clinical practice [6,23]. While methotrexate was also the principal anchor drug in our study population, combination therapy-particularly dual csDMARD regimens-was more commonly employed than methotrexate monotherapy. This preference for combination therapy aligns with findings reported by Siddiqui et al., who similarly documented higher rates of dual therapy over monotherapy in patients with rheumatoid arthritis [23]. While Bawazir et al. reported higher use of biologics [12], the present study observed greater reliance on cs-DMARDs, likely reflecting differences in physician prescribing practices, patient affordability, and the effectiveness of cs-DMARD mono or combination therapy in achieving low disease activity in a predominantly low socioeconomic cohort. Among the nonsteroidal anti-inflammatory drugs (NSAIDs), indomethacin was most used, followed by naproxen and etoricoxib. A substantial proportion of the study cohort was receiving corticosteroid therapy, indicating that most patients were likely experiencing active inflammatory disease necessitating steroid use for pain and inflammation control.

In the present study, we conducted a short-term follow-up to evaluate outcomes based on the treatment patterns to which patients adhered. At baseline, only a small proportion of patients were in remission (2.48%) or had low disease activity, while the majority presented with moderate disease activity (66.94%), and a considerable number had high disease activity (23.14%). By the end of three months, there was a marked improvement, with a sharp decline in the proportion of patients with high disease activity. Remission and low disease activity rates increased notably, while moderate disease activity showed a substantial reduction. These findings reflect a significant reduction in overall disease activity over the study period, aligning with the expected therapeutic onset of disease-modifying antirheumatic drugs (DMARDs), which typically require two to three months to demonstrate clinical efficacy.

This study is limited by its relatively small sample size, single-center design, short-term follow-up, and loss of follow-up, which may affect the generalizability of the findings. Additionally, the tertiary care setting may introduce selection bias, as it typically involves patients with more severe or complex disease profiles.

## Conclusions

Rheumatoid arthritis predominantly affects middle-aged individuals and females, with common clinical features including arthralgia, early morning stiffness, and polyarticular joint involvement. The wrist joint was most frequently affected, followed by the PIP and MCP joints. Extra-articular manifestations were common, with anemia being the most frequent. Obesity was the most prevalent comorbidity. Combination therapy with conventional synthetic DMARDs, particularly methotrexate and hydroxychloroquine, was the most common treatment approach. Indomethacin was the predominant NSAID used, and a significant number of patients were also on corticosteroids. Over a three-month period, there was a notable reduction in disease activity, with increased rates of remission and low disease activity, consistent with the expected therapeutic response time of DMARDs. These findings underscore the need for early RA diagnosis and tailored treatment strategies in Central India, particularly for middle-aged women with polyarticular disease. Larger, multicenter studies with longer follow-ups are needed to validate these findings and assess long-term outcomes.
